# Correction: Magnitude and determinants of improved household latrine utilization in Ethiopia: Multilevel analysis of the mini Ethiopian Demographic Health Survey (EDHS) 2019

**DOI:** 10.1371/journal.pone.0327043

**Published:** 2025-06-25

**Authors:** Aragaw Tesfaw, Mulu Tiruneh, Melkalem Mamuye, Zebader Walle, Wondwosen Teshager, Fentaw Teshome, Alebachew Taye, Wondimnew Dessalegn, Gashaw Walle, Asaye Alamneh Gebeyehu

The fourth and tenth authors name are spelled incorrectly. The correct names are: Wondwosen Teshager, Asaye Alamneh Gebeyehu.
