# Supplementary material for: Magnitude and determinants of improved household latrine utilization in Ethiopia: Multilevel analysis of the mini Ethiopian Demographic Health Survey (EDHS) 2019
Source: PLoS One. 2023 Aug 2;18(8):e0289427. doi: 10.1371/journal.pone.0289427 (PMC10395897; doi:10.1371/journal.pone.0289427)
Supplement: Attachment — Submitted filename: Response to reviewers.docx [file pone.0289427.s002.docx]

Thank you very much for all the comments provided regarding our manuscript entitled “Magnitude and Determinants of improved household latrine Utilization in Ethiopia: Multilevel Analysis of the Mini Ethiopian Demographic Health Survey (EDHS) 2019” which are fully accepted and included in the revised version. I have accordingly made necessary revisions on the paper following the comments provided from the reviewer and editor. For your kind consideration, please find a point-by-point response to the comments and a submitted new revised version of the manuscript. All new changes have been highlighted in dark blue in the main document in order to facilitate review.

I hope that you will find the edits as per your expectation and I look forwards to hear from you

Please kindly find our point-by-point response for the comments provided.

**Response to academic editor comments**

Journal Requirements:

When submitting your revision, we need you to address these additional requirements.

1. Please ensure that your manuscript meets PLOS ONE's style requirements, including those for file naming. The PLOS ONE style templates can be found at
   <https://journals.plos.org/plosone/s/file?id=wjVg/PLOSOne_formatting_sample_main_body.pdf> and 
   <https://journals.plos.org/plosone/s/file?id=ba62/PLOSOne_formatting_sample_title_authors_affiliations.pdf>

**Author response:** We prepared our manuscript using PLOS ONE's styles /templates

2. We noticed you have some minor occurrence of overlapping text with the following previous publication(s), which needs to be addressed:
   - <https://doi.org/10.1371/journal.pone.0241270>
   In your revision ensure you cite all your sources (including your own works), and quote or rephrase any duplicated text outside the methods section. Further consideration is dependent on these concerns being addressed.

**Author response:** We have now modified the document based on the comments provided by the editor in the main document

3. In your Data Availability statement, you have not specified where the minimal data set underlying the results described in your manuscript can be found. PLOS defines a study's minimal data set as the underlying data used to reach the conclusions drawn in the manuscript and any additional data required to replicate the reported study findings in their entirety. All PLOS journals require that the minimal data set be made fully available. For more information about our data policy, please see <http://journals.plos.org/plosone/s/data-availability>.

"Upon re-submitting your revised manuscript, please upload your study’s minimal underlying data set as either Supporting Information files or to a stable, public repository and include the relevant URLs, DOIs, or accession numbers within your revised cover letter. For a list of acceptable repositories, please see <http://journals.plos.org/plosone/s/data-availability#loc-recommended-repositories>. Any potentially identifying patient information must be fully anonymized.

Important: If there are ethical or legal restrictions to sharing your data publicly, please explain these restrictions in detail. Please see our guidelines for more information on what we consider unacceptable restrictions to publicly sharing data: <http://journals.plos.org/plosone/s/data-availability#loc-unacceptable-data-access-restrictions>. Note that it is not acceptable for the authors to be the sole named individuals responsible for ensuring data access.

We will update your Data Availability statement to reflect the information you provide in your cover letter.

**Author response:** The data for this study will be obtained from the cross-ponding author on a reasonable request

4. Please amend the manuscript submission data (via Edit Submission) to include author Asaye Alemneh.

**Author response:** We have included author Asaye Alemneh in the submission link based on the comments

5. Your ethics statement should only appear in the Methods section of your manuscript. If your ethics statement is written in any section besides the Methods, please move it to the Methods section and delete it from any other section. Please ensure that your ethics statement is included in your manuscript, as the ethics statement entered into the online submission form will not be published alongside your manuscript.

**Author response**: We have now put the ethics statement in the methods section of the main document based on the comments

6. We note you have included a table to which you do not refer in the text of your manuscript. Please ensure that you refer to Table 4 and 5 in your text; if accepted, production will need this reference to link the reader to the Table.

[Note: HTML markup is below. Please do not edit.]
**Author response**: We have corrected/ modified in the main document based on the comments

**Response to reviewer comments**

Subject: Reviewer comment for Research Article PONE-D-22-34120

Thank you very much for the opportunity given for me to review the manuscript entitled "Magnitude and Determinants of improved household latrine Utilization in Ethiopia: Multilevel Analysis of the Mini Ethiopian Demographic Health Survey (EDHS) 2019."

As mentioned, the authors did a secondary data analysis that used to determine factors associated with an improved latrine Utilization based on the mini–Ethiopian Demographic Health Survey (EDHS) 2019 data set. Given that, I forwarded my comments that I believed which could help authors to improve the quality of their manuscript.

**General comment:**

Overall, despite the known limitation, this study may provide complementary evidence related to latrine Utilization in Ethiopia. I think, the manuscript need language revision and it would be good to refer similar research articles and or tools used for secondary data analysis and follow the writing styles of standard (peer reviewed) published articles. There is mix-up of words and or phrases that are commonly used to report primary studies. For example, phrases such as “This **study was conducted in Ethiopia”** may give wrong impression and it seems this study was designed to undertake independent study that collect primary research data**.** Hence, I suggest the authors should revise the document thinking as you are using a secondary data that was collected as part of the national survey.

**Author response:** We would like to thank and appreciate the reviewer for providing important comments and suggestions**.** We have now modified the document based on the comments provided by the reviewer in the main document after reviewing some similar research articles in the area.

**Specific comments**

**#Introduction:** Though the objective of the current study is stated in the abstract, the rationale and aim of the study is not clearly explained in the main document (introduction). Indeed, at the end of the last paragraph the author stated, “The findings of this study will help to increase utilization of improved latrines in the households of Ethiopia**”.** Can you please justify how? Rather, such type of studies often used to generate further and stronger information that can complement the original survey report.

Please look at the national EDHS report that was published in May 2021 and articulate the objectives of your study to fil gaps that is not addressed in the report-Perhaps it is still possible to develop testable hypothesis based on your interest of outcome and the main exposure variable. It would be also good to present the conceptual framework that can explain the theoretical and logical relationship of study variables included in this study.

**Author response**: We accepted the reviewer comments and suggestions and we have now corrected/ modified in the main document based on the comments

**Method:** brief description of study settings, design and sampling procedures of the survey might be important but that should not be more detailed. Instead, you can cite the published report and explain the structure and composition of the main dataset related to the main outcome variable and related factor variables used for this study. Similarly, instead of describing in detail the general approaches (methods) used to conduct the survey, it is better to provide enough information on the techniques and procedures on how you extract the study variables and data used to achieve your main objective.

From my reading (both the manuscript and EDHS survey report), I understood that authors included all eligible samples (households) who were participated in the 2019 mini–Ethiopian Demographic Health Survey. That might be Ok to include all participants, but a systematic sampling (selection) of study units using predefined criteria would be preferred. Meaning, once the maximum power is achieved for the specific objective, the authors should further consider quality of survey data. Not the sample size but such type of dataset requires advanced and sound data handling and management - that is mandatory while using secondary data analysis.

Similarly, it is not the mathematical formula and or the general assumption (principles) rather authors should clearly explain their own practical analysis techniques and the respective outputs- that used to decide the next procedure.

I think, definitions of terms should precede data management and analysis- not at the end of research methods.

**Author response**: We have corrected/ modified at the main document based on the comments

**Results**: like that of the method, the descriptive part of your result section reflected the setting and background characteristics of survey participant- it seems the original report. It is not focused as that was expected from secondary data analysis studies.

It is not clear how and where the X^2^-test and the corresponding p-vale presented in Table-1 were generated while only the frequency and percentage of one-dimensional variables are presented- which is not cross-tabulated.

In general, the descriptive findings and the whole results lack the standard criteria used for data visualization (presentation)- perhaps the results (Tabular presentation) reflect the methodological limitation and I felt authors should give more emphasis for data management. For instance, at least those important variables should be recoded or transformed using simple methods and then the reader could grasp the key findings related to this study.

It is still difficult to understand the findings generated from the multilevel analysis (Modelling). It is not supported with descriptive reports that can provide additional information- If not mistaken I did not see any descriptive findings or estimates that compare the observed frequency related with the main outcome (Improved Vs Unimproved). I suggest either to modify the descriptive findings or add columns to include the observed frequencies [n (%)] in the multilevel analysis (Table 5).

I think, authors should provide their response and explanation to clarify the above issues and then we can proceed to the next section.

**Author response**: We have corrected/ modified at the main document based on the comments. We add descriptive statistical analysis results in the revised document based on the reviewer suggestions.
