# Supplementary material for: Magnitude and determinants of improved household latrine utilization in Ethiopia: Multilevel analysis of the mini Ethiopian Demographic Health Survey (EDHS) 2019
Source: PLoS One. 2023 Aug 2;18(8):e0289427. doi: 10.1371/journal.pone.0289427 (PMC10395897; doi:10.1371/journal.pone.0289427)
Supplement: Attachment — Submitted filename: Comments for Manuscript Number PONE-D-22-34120.docx [file pone.0289427.s004.docx]

Comments for Manuscript Number:  PONE-D-22-34120

*Title:*  Magnitude and Determinants of improved household latrine Utilization in Ethiopia: Multilevel Analysis of the Mini Ethiopian Demographic Health Survey (EDHS) 2019

*General remarks:*

This paper evaluated Magnitude and Determinants of improved household latrine Utilization in Ethiopia, which are interesting information to capture. The manuscript can be accepted for publication after some revisions as below:

Background:

- Please clarify and provide references and clear examples related to “inconsistences between the study’s findings” in the sentence “However, prior studies on the issue are limited and outdated or focused on specific geographical regions in the country, moreover there are inconsistences between the study’s findings”

Results:

- When compare with previous studies, the authors should have one or two sentences that discussed the similarity or consistent results of this study and previous studies. How consistent or similar?
- Avoid using too much “The odds of”. You should use only one time.
- When discuss the “The variation of the fitted models after Multilevel Analysis”, need to assess which model is the most reliable and can be applied for other similar studies.
- Please mention that sex and age are not important factors. Is it consistent with previous studies?
- Grammar errors: This because these are the big administrative cities in the country and the households in these cities have access to using an improved latrine facility as compared to other regions in the country; Determinate of improved Latrine utilization in Ethiopia, etc.

Conclusion

- English improvement should be conducted.
- There should be included a number found from the result for low utilization of improved latrine.
- In this part, the most recommended model for data analysis should be added.
- The limitation and recommendations for future research and suggestion for more variables during the cross-country census survey for better understanding.

*Conclusion:* The paper had good information, however, it needs some revisions and improvement.

🡪 Accepted with minor changes.
