# Supplementary material for: Magnitude and determinants of improved household latrine utilization in Ethiopia: Multilevel analysis of the mini Ethiopian Demographic Health Survey (EDHS) 2019
Source: PLoS One. 2023 Aug 2;18(8):e0289427. doi: 10.1371/journal.pone.0289427 (PMC10395897; doi:10.1371/journal.pone.0289427)
Supplement: Attachment — Submitted filename: Response to reviewers.docx [file pone.0289427.s005.docx]

Thank you very much for all the comments provided regarding our manuscript entitled “Magnitude and Determinants of improved household latrine Utilization in Ethiopia: Multilevel Analysis of the Mini Ethiopian Demographic Health Survey (EDHS) 2019” which are fully accepted and included in the revised version. I have accordingly made necessary revisions on the paper following the comments provided from the reviewer and editor. For your kind consideration, please find a point-by-point response to the comments and a submitted new revised version of the manuscript. All new changes have been highlighted in blue in the main document in order to facilitate review.

I hope that you will find the edits as per your expectation and I look forwards to hear from you

Please kindly find our point-by-point response for the comments provided.

**Author response to reviewer I comments**

Reviewers' comments:

Reviewer #1: Overall, the authors addressed most of my previous comments and noted a significant improvement on the revised version. Hence, I believed the manuscript can fulfill the required criteria for publication with minor modification/ revision.
Therefore, appreciating the author’s commitment, who made further/ major revision and provided satisfactory responses, I would like to remind some of the previous comments, and I think it is better to look the paper once again before production/publication. Hope, you can still improve the language and other editorial/formatting

Method and result section

Do you mean you included all eligible samples (households) and variables from 2019 mini-EDHS? I think, you included all participates (HH) but only selected variable based on your specific objective. If that is the case; it should be stated as “We include all eligible samples (households) who were participated in the 2019 mini–Ethiopian Demographic Health Survey based on predefined selection criteria (study variables) related to the specific objectives.
If you used the full dataset with different/ advanced analytical approaches, that is ok and you clearly described the statistical analysis procedure- though you can still make further data management such as creating new variables or recoding old variables.

**Author response:** Thank you very much the reviewer for the constructive comments provided. As the reviewer said, in our analysis, we used the “HR data set” **based on the guideline of DHS program statistics** and the study population were household members who have used latrine in the past five year of the survey. A weighted sample of 8663 individuals were involved in the analysis. Therefore we accepted the comment and we included the suggested statement form the reviewer i.e. “We include all eligible samples (households) who were participated in the 2019 mini–Ethiopian Demographic Health Survey based on predefined selection criteria (study variables) related to the specific objectives”.

• For instance, Number of households member sharing toilet can be recoded in to two/three categories (e.g. < 5 and above) and then you can explain the procedure within data management section.

**Author response:** We have corrected based on the comment in the main document as suggested by the reviewer. We categorized number of household’s member sharing toilet as < 5 and ≥ 5 household members.

• In table- 2: remove Type of toilet facility from the last row as it is a duplication of the second variable with merged categories. If interested to present findings for each type of latrine, you can illustrate using separate graphs or chart

**Author response:** We have corrected based on the comment in the main document as suggested by the reviewer.

• As to my knowledge, simple statistical tests such as X2 and T- test are used to assess or determine the relationship or association between two or more variables – like that of table-4. While there is only one one-dimensional variables in Table- 1. Can you please justify the importance of are X2 - test and the corresponding P- value? If not better to delete the last two columns in table-1

**Author response:** We have corrected based on the comment in the main document as suggested by the reviewer. As you have said, x2 test is just used for just measuring the association between categorical variables and it will be used as also the first step to run logistic regression model since it is one of the assumptions. But in our study we used it just for showing the association between each individual and community level factors with the outcome variable. Thank you for giving your time to review our paper for improvements

**Author response to reviewer -2**

*Title:*  Magnitude and Determinants of improved household latrine Utilization in Ethiopia: Multilevel Analysis of the Mini Ethiopian Demographic Health Survey (EDHS) 2019

*General remarks:*

This paper evaluated Magnitude and Determinants of improved household latrine Utilization in Ethiopia, which are interesting information to capture. The manuscript can be accepted for publication after some revisions as below:

Background:

- Please clarify and provide references and clear examples related to “inconsistences between the study’s findings” in the sentence “However, prior studies on the issue are limited and outdated or focused on specific geographical regions in the country, moreover there are inconsistences between the study’s findings”

**Author response:** thank you for the reviewer for the constructive comments provided. We have corrected the comments in the main document

Results:

- When compare with previous studies, the authors should have one or two sentences that discussed the similarity or consistent results of this study and previous studies. How consistent or similar?

**Author response:** thank you again to the reviewer for the constructive comments provided. We have tried to explain the similarities or the consistence’s between our study and other study findings in the main document

- Avoid using too much “The odds of”. You should use only one time.

**Author response:** We have corrected in the main document

- When discuss the “The variation of the fitted models after Multilevel Analysis”, need to assess which model is the most reliable and can be applied for other similar studies.

**Author response:** thank you for the reviewer for the comments the provided. Regarding the models, we already discussed about in the methods section. In multilevel analysis due to the hierarchical nature of the data, we have fitted four different models. The first model was the null model which is an empty model developed without individual or community level variables and the second model (Model II) contained the effects of the individual-level variables on the response variable. The third model (Model III) included the influences of the community-level variables on the response variable, and the final model examined the effects of individual-level and community-level variables. The best model to be used in this case is the model which has the lower deviance statistics in relative comparisons between the models. So, a model with a lower deviance (a measure of model fitness) value was considered as the better-fitted model for this study. Therefore, the final model was the best model for our study.

- Please mention that sex and age are not important factors. Is it consistent with previous studies?

**Author response:** there are some studies which have found statistical significant association between sex, and age with latrine utilization. These studies are single area studies and most are conducted using a small size. In our study the data was collected at the household level as a national population based survey and age and sex data was taken from the head of the households.

- Grammar errors: This because these are the big administrative cities in the country and the households in these cities have access to using an improved latrine facility as compared to other regions in the country; Determinate of improved Latrine utilization in Ethiopia, etc.

**Author response:** we have corrected/edited in the main document

Conclusion

- English improvement should be conducted.
- There should be included a number found from the result for low utilization of improved latrine.
- In this part, the most recommended model for data analysis should be added.

**Author response:** Thank you the reviewer for all the constrictive comments provided. We have corrected/edited in the main document. As mentioned in the methods section, the best recommended model for our study is the final model which was developed from both individual and community level factors and it has the lower deviance statistics.

- The limitation and recommendations for future research and suggestion for more variables during the cross-country census survey for better understanding.

**Author response:** Thank you the reviewer for all the constrictive comments provided. We have corrected/edited in the main document. We recommend further research to be done at multicounty level survey data i.e. by including Demographic health survey data’s of several countries for making comparisons. In addition further studies should be done also on the full EDHS data set when it is released.

*Conclusion:* The paper had good information, however, it needs some revisions and improvement.

**Author response:** Thank you for giving your time to review our paper for improvements
