# Supplementary material for: Magnitude and determinants of improved household latrine utilization in Ethiopia: Multilevel analysis of the mini Ethiopian Demographic Health Survey (EDHS) 2019
Source: PLoS One. 2023 Aug 2;18(8):e0289427. doi: 10.1371/journal.pone.0289427 (PMC10395897; doi:10.1371/journal.pone.0289427)
Supplement: Attachment — Submitted filename: Reviewer comments (HT) _ PONE-D-22-34120.docx [file pone.0289427.s001.docx]

Date:	January 29, 2022

To:	PLOS ONE

Subject: Reviewer comment for Research Article PONE-D-22-34120

Dear Dr Gizat and All

Thank you very much for the opportunity given for me to review the manuscript entitled "Magnitude and Determinants of improved household latrine Utilization in Ethiopia: Multilevel Analysis of the Mini Ethiopian Demographic Health Survey (EDHS) 2019."

As mentioned, the authors did a secondary data analysis that used to determine factors associated with an improved latrine Utilization based on the mini–Ethiopian Demographic Health Survey (EDHS) 2019 data set. Given that, I forwarded my comments that I believed which could help authors to improve the quality of their manuscript.

**General comment:**

Overall, despite the known limitation, this study may provide complementary evidence related to latrine Utilization in Ethiopia. I think, the manuscript need language revision and it would be good to refer similar research articles and or tools used for secondary data analysis and follow the writing styles of standard (peer reviewed) published articles. There is mix-up of words and or phrases that are commonly used to report primary studies. For example, phrases such as “This **study was conducted in Ethiopia”** may give wrong impression and it seems this study was designed to undertake independent study that collect primary research data**.** Hence, I suggest the authors should revise the document thinking as you are using a secondary data that was collected as part of the national survey.

**Specific comments**

**#Introduction:** Though the objective of the current study is stated in the abstract, the rationale and aim of the study is not clearly explained in the main document (introduction). Indeed, at the end of the last paragraph the author stated, “The findings of this study will help to increase utilization of improved latrines in the households of Ethiopia**”.** Can you please justify how? Rather, such type of studies often used to generate further and stronger information that can complement the original survey report.

Please look at the national EDHS report that was published in May 2021 and articulate the objectives of your study to fil gaps that is not addressed in the report-Perhaps it is still possible to develop testable hypothesis based on your interest of outcome and the main exposure variable. It would be also good to present the conceptual framework that can explain the theoretical and logical relationship of study variables included in this study.

**Method:** brief description of study settings, design and sampling procedures of the survey might be important but that should not be more detailed. Instead, you can cite the published report and explain the structure and composition of the main dataset related to the main outcome variable and related factor variables used for this study. Similarly, instead of describing in detail the general approaches (methods) used to conduct the survey, it is better to provide enough information on the techniques and procedures on how you extract the study variables and data used to achieve your main objective.

From my reading (both the manuscript and EDHS survey report), I understood that authors included all eligible samples (households) who were participated in the 2019 mini–Ethiopian Demographic Health Survey. That might be Ok to include all participants, but a systematic sampling (selection) of study units using predefined criteria would be preferred. Meaning, once the maximum power is achieved for the specific objective, the authors should further consider quality of survey data. Not the sample size but such type of dataset requires advanced and sound data handling and management - that is mandatory while using secondary data analysis.

Similarly, it is not the mathematical formula and or the general assumption (principles) rather authors should clearly explain their own practical analysis techniques and the respective outputs- that used to decide the next procedure.

I think, definitions of terms should precede data management and analysis- not at the end of research methods.

**Results**: like that of the method, the descriptive part of your result section reflected the setting and background characteristics of survey participant- it seems the original report. It is not focused as that was expected from secondary data analysis studies.

It is not clear how and where the X^2^-test and the corresponding p-vale presented in Table-1 were generated while only the frequency and percentage of one-dimensional variables are presented- which is not cross-tabulated.

In general, the descriptive findings and the whole results lack the standard criteria used for data visualization (presentation)- perhaps the results (Tabular presentation) reflect the methodological limitation and I felt authors should give more emphasis for data management. For instance, at least those important variables should be recoded or transformed using simple methods and then the reader could grasp the key findings related to this study.

It is still difficult to understand the findings generated from the multilevel analysis (Modelling). It is not supported with descriptive reports that can provide additional information- If not mistaken I did not see any descriptive findings or estimates that compare the observed frequency related with the main outcome (Improved Vs Unimproved). I suggest either to modify the descriptive findings or add columns to include the observed frequencies [n (%)] in the multilevel analysis (Table 5).

I think, authors should provide their response and explanation to clarify the above issues and then we can proceed to the next section.
